# Effectiveness of Functional Electrical Stimulation in Improving Clinical Outcomes in the Upper Arm following Stroke: A Systematic Review and Meta-Analysis

**DOI:** 10.1155/2015/729768

**Published:** 2015-01-22

**Authors:** Amir K. Vafadar, Julie N. Côté, Philippe S. Archambault

**Affiliations:** ^1^School of Physical and Occupational Therapy, McGill University and Interdisciplinary Research Center in Rehabilitation (CRIR), 3654 Promenade Sir William Osler, Montreal, QC, Canada H3G 1Y5; ^2^Department of Kinesiology and Physical Education, McGill University and Interdisciplinary Research Center in Rehabilitation (CRIR), 475 Pine Avenue West, Montreal, QC, Canada H2W 1S4

## Abstract

*Background.* Different therapeutic methods are being used to prevent or decrease long-term impairments of the upper arm in stroke patients. Functional electrical stimulation (FES) is one of these methods, which aims to stimulate the nerves of the weakened muscles so that the resulting muscle contractions resemble those of a functional task.* Objectives.* The objective of this study was to review the evidence for the effect of FES on (1) shoulder subluxation, (2) pain, and (3) upper arm motor function in stroke patients, when added to conventional therapy.* Methods.* From the 727 retrieved articles, 10 (9 RCTs, 1 quasi-RCT) were selected for final analysis and were rated based on the PEDro (Physiotherapy Evidence Database) scores and the Sackett's levels of evidence. A meta-analysis was performed for all three considered outcomes.* Results.* The results of the meta-analyses showed a significant difference in shoulder subluxation in experimental groups compared to control groups, only if FES was applied early after stroke. No effects were found on pain or motor function outcomes.* Conclusion.* FES can be used to prevent or reduce shoulder subluxation early after stroke. However, it should not be used to reduce pain or improve upper arm motor function after stroke.

## 1. Introduction

Stroke is a global health-care problem that is both serious and disabling [1]. In high-income countries, stroke is the third most common cause of death and is the main cause of acquired adult impairment [1]. As most patients with stroke survive the initial injury, the biggest effect on patients and families is usually through long-term impairment. It has been shown that about 40% of people who survive a stroke still have significantly impaired function in their affected arm after 3 months, whereas 40% have mild to moderate impairments and only 20% have entirely normal function [2]. Motor impairment in the arm persisting for a long time can make patients become functionally dependent on others for everyday activities. In addition, this can lead to other complications such as shoulder subluxation and pain.

Shoulder subluxation is a major challenge in the rehabilitation of stroke patients. It may not only affect the upper limb treatment process, but can also lead to additional complications such as pain, which can further delay the recovery of function. The reported incidence of shoulder subluxation as one of the major consequences of motor dysfunction varies in stroke patients, from 17% [3] to 81% [4]. Traditionally, shoulder subluxation has been described as inferior subluxation [5, 6], meaning that, in a hemiplegic shoulder, gravitational forces generated by the weight of the arm pull the head of the humerus downward because the supraspinatus muscle and posterior deltoid, which are key components in counteracting this downward pull, are weak or paralyzed [7].

Shoulder pain is another impairment that can interfere with the recovery of patients after stroke. Hanger and colleagues [8] suggested that the cause of pain in people recovering from a stroke may be multifactorial and that these factors may further vary at different stages of recovery. For example if pain occurs during the flaccidity phase and is associated with shoulder subluxation, it is likely caused by excessive stretches and associated damages to the soft tissues (capsule, ligaments, and muscles) around the shoulder [9]; but if it occurs during the spastic phase of recovery, associated with persistence of spasticity patterns (adduction and internal rotation) in the shoulder, it may be due to the shortening of capsule and ligaments and possible muscle contractures [10]. The speed of motor recovery has also been shown to be associated with shoulder pain. Malouin et al. [11] suggest that patients with slow recovery of motor function in the affected arm tend to develop joint pain whereas patients with faster motor function recovery do not experience the same degree of difficulties. Therefore, enhancement of motor function is crucial in the rehabilitation of stroke patients as it may prevent or decrease the incidence of pain and subluxation in the shoulder joint.

Different therapeutic techniques have been employed for the treatment of arm motor function in hemiplegia. Functional electrical stimulation (FES) is one of these methods that consists in using electrical currents in stimulating the nerves connected to the paralyzed muscles in precise sequence and magnitude so that the outcome resembles functional tasks [12]. In fact, FES aims to generate movements or functions, which mimic normal voluntary movements, and therefore to restore the functions served by those movements [13]. In the shoulder joint, FES is mainly used to stimulate those muscles that are responsible to maintain the head of the humerus in the glenoid fossa (especially the supraspinatus and the posterior deltoid which counteract the inferior displacement of the humerus [7]) and can therefore prevent or restore subluxation, reduce pain, and improve function. FES has some specific characteristics that make it distinct from other forms of electrical stimulation. The frequency range of FES falls between 10 and 50 HZ [14] and it directly stimulates the nerves or their motor points, not the muscle fibers. Moreover, compared to other forms of electrical stimulation devices, FES can be used to elicit electrical stimulation in a specific sequence and magnitude, which can be used to create the muscle activity required for the performance of a functional task [12].

In 2002, Ada and Foongchomcheay [15] conducted a meta-analysis on the effect of electrical stimulation on shoulder outcomes after stroke. They showed that FES was superior to conventional therapy alone in the treatment of shoulder subluxation and arm motor function but was not effective in the treatment of pain early after stroke. Since 2002 more studies have been published which have examined the effectiveness of FES on shoulder outcomes after stroke, especially on motor function and pain. Therefore we performed this systematic review and meta-analysis to address those new findings. In addition, unlike the meta-analysis by Ada and Foongchomcheay, which was done based on shoulder subluxation as the primary outcome and pain and motor function as secondary outcomes, we gave the same weight to each outcome in our database searches so that we did not miss the studies which had only examined one or two of the three outcomes. Accordingly, the specific objective for this review was to estimate the extent to which FES impacts on shoulder subluxation, pain, and upper arm motor function in stroke patients. The primary question of this review in PICO format (population, intervention, comparison, and outcome) is: “In stroke patients, is FES effective in reducing shoulder subluxation and pain and in improving arm motor function, as compared to conventional therapy alone?” The three main outcomes of this review were shoulder subluxation, shoulder pain and motor function in the upper arm.

## 2. Methods

### 2.1. Eligibility Criteria

#### 2.1.1. Types of Studies

All randomized controlled trials (RCTs) and quasi-RCTs and controlled trials examining the effect of FES on shoulder and upper arm outcomes were considered for this review. No language, publication date, or publication status restrictions were imposed to our database searches.

#### 2.1.2. Types of Participants

Trials were considered if they included patients of any age or gender with a clinical diagnosis of stroke, either ischemic or hemorrhagic. Accordingly, trials that included patients with other neurological conditions leading to hemiplegia (e.g., head injury) were not included.

#### 2.1.3. Types of Intervention

To be included in the review, the type of electrical stimulation, whether called FES, neuromuscular electrical stimulation (NMES), or simply electrical stimulation, had to contain the characteristics of a typical FES, that is, stimulations that had a frequency of 10 to 50 HZ (typical frequency range of FES [14]). Only surface electrical stimulations were considered for this review.

#### 2.1.4. Types of Outcome Measures

The three main outcome measures of this review were shoulder subluxation, shoulder pain, and motor function in the upper arm.

### 2.2. Search Strategy

Bibliographic databases of MEDLINE, CINHAL, EMBASE, Cochrane, PEDro (Physiotherapy Evidence Database), and PsycINFO were searched, from the beginning and up to May 2014 inclusively, without any language restriction and with the following keywords:* electrical stimulation, functional electrical stimulation, neuromuscular electrical stimulation, FES, NMES, shoulder joint, subluxation, pain, motor function, stroke and hemiplegia *(Table 1). Relevant studies were identified from titles and abstracts. In addition, reference lists of retrieved articles were reviewed to identify other relevant studies.

### 2.3. Study Selection

**Figure 1:**
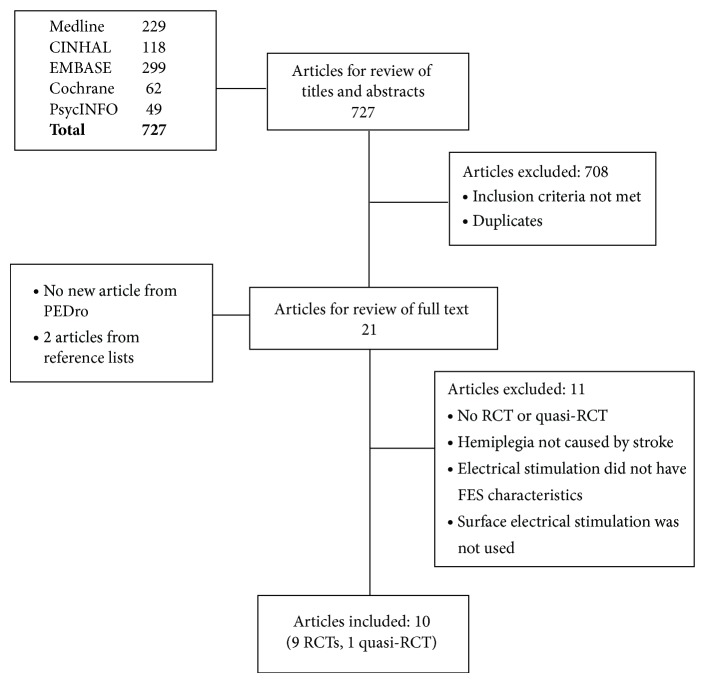
Flowchart of database search and article selection process.

Database searching was performed by two evaluators. Each database search resulted in a number of appropriate articles. The abstracts of all these articles were reviewed and if the abstract was not available, then the full text was obtained. After examining the abstracts, 21 unique articles were found to be potentially appropriate for the review. The full text of all these 21 articles was obtained and studied. Ultimately, based on the predefined inclusion and exclusion criteria, 10 articles (9 RCTs and 1 quasi-RCT) were selected for the final analysis (Figure 1). Two articles that were written in a language other than English (one Korean and one Turkish) were translated.

### 2.4. Data Collection Process

For each outcome, we qualitatively reviewed each article and performed a meta-analysis. To perform the meta-analysis, mean and standard deviations of each outcome measure after treatment were pooled. If median and minimum/maximum were reported, data was converted to mean and standard deviation using the formulae described by Hozo et al. [16]. If mean and standard deviations were not reported, the data from the meta-analysis by Ada and Foongchomcheay [15] was used. If median and interquartile range were reported, mean and standard deviations were estimated using the formula explained in Rodbard [17], assuming that the data were normally distributed. In the meta-analyses, effect sizes were reported as standard mean differences using Hedges's *g*. The results of heterogeneity (*χ* ^2^) and consistency (*I* ^2^) tests as well as the test for the overall effect were also reported. If the measurement of the outcomes were similar across studies, then a fixed effect model was used; and when they were not similar, a random effect model was used. The power of each meta-analysis was calculated retrospectively using the formulae explained by Valentine et al. [18], considering the observed effect, number of studies, and average within-study sample size. The meta-analyses were performed using Review Manager (RevMan) software, version 5.2 [19].

To assess the risk of bias across studies, we evaluated each retrieved article and identified any drop-outs or missing data as reported by the authors. In addition, for all three outcomes, we divided the articles as being conducted either early or late after stroke. We chose a cut-off of 6 months for early/late, as it has been observed that motor function recovery is most rapid during the first month after stroke, becomes slow during subsequent months, and eventually goes to a plateau phase by 6 months after stroke [20]. Therefore we separated the acute and subacute stages (before 6 months) from the chronic stage (after 6 months), as the chance of recovery during the chronic stage is lower. Finally, for the subluxation outcome, in addition to investigating the short-term effects of FES therapy by evaluating the changes at the end of the intervention period, we also estimated the long-term effects by performing a separate qualitative evaluation on those studies that carried out a follow-up assessment after the cessation of treatment.

### 2.5. Risk of Bias in Each Individual Study

The risk of bias in each individual study was examined based on the PEDro Scale developed by the Center for Evidence Based Practice in Australia [21, 22]. The PEDro Scale has shown moderate to high levels of interrater reliability (Intraclass Correlation Coefficient (ICC) 0.54–0.91; [21]). With the PEDro Scale, the following indicators of methodological rigor were scored independently as either absent or present (0 or 1): (1) specification of eligibility criteria, (2) random allocation, (3) concealed allocation, (4) prognostic similarity at baseline, (5) subject blinding, (6) therapist blinding, (7) assessor blinding, (8) greater than 85% follow-up for at least 1 key outcome, (9) intention-to-treat analysis, (10) between-group statistical analysis for at least 1 key outcome, and (11) point estimates of variability provided for at least 1 key outcome. According to the PEDro guidelines, criteria (2) through (11) are used for scoring purposes so that a score from 0 to 10 can be obtained [23]. Studies scoring 9 or 10 were rated as excellent, 6 to 8 as good, 4 to 5 as fair, and lower than 4 as poor [24].  Table 2 shows the PEDro scores for the 10 included articles.

In addition to the PEDro scale, the quality of the studies was rated according to the Sackett's levels of evidence [25], which was adapted to include PEDro scaling. According to this quality assessment method, evidence level of 1a (strong) was given when two or more high quality RCTs were showing similar results (PEDro ≥ 6); evidence level of 1b (moderate) was given when at least one RCT of high quality was found (PEDro = 6); evidence level of 2a (limited) was given when at least one fair quality RCT was found (PEDro 4-5); evidence level of 2b (limited) was given when at least one poor quality RCT or well-designed nonexperimental study was found (PEDro < 4); evidence level of 3 (consensus) was given when a number of pre-post studies showed similar results or there was an agreement by an expert panel; evidence level of 4 (conflict) was given when there was a conflicting evidence of two or more equally well designed studies; finally, evidence level of 5 was given when no well-designed study was found.

## 3. Results

Table 2 summarizes the findings of the 10 selected empirical studies and shows the quality score for each trial. The highest PEDro score was 8 [34] and the lowest was 3 [26]. The highest evidence level was 1a and the lowest was 3. Nine of the selected articles were RCTs and one was a quasi-RCT.

### 3.1. Subluxation

Seven out of ten articles (Wang et al. [30], Kim et al. [28], Kobayashi et al. [26], Linn et al. [29], Baker and Parker [32], Koyuncu et al. [27], and Faghri et al. [33]) estimated shoulder subluxation as an outcome measure. Two studies (Wang et al. and Kim et al.) reported two categories of participants based on the time after stroke, either early or late; therefore they were considered as two separate studies (Wang-E, Wang-L and Kim-E, and Kim-L). In addition, another study divided the participants based on the muscle that was stimulated (deltoid or supraspinatus) and was therefore considered as two separate studies (Kobayashi-D and Kobayashi-S [26]). In total, ten articles were considered for the review. The retrieved articles were then divided into two groups based on the onset of stroke, early and late.

In all of the ten retrieved articles, FES was applied in addition to conventional physical and/or occupational therapy. In all studies, the control group received the same treatment as the study group except for the FES treatment. Subjects remained passive during the FES treatment sessions; they did not perform any activity or receive any other form of treatment while FES was administered. The measurement of shoulder subluxation in all experiments was done using an antero-posterior radiographic X-ray and the displacement of the head of the humerus was measured in cm or mm.

#### 3.1.1. Early Treatment

**Figure 2:**
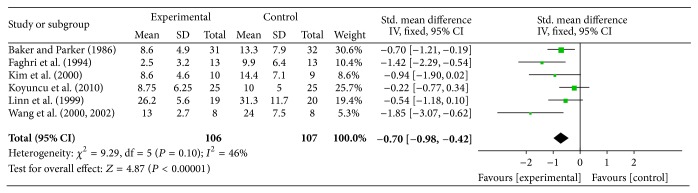
Effect of FES on shoulder subluxation “early” after stroke, with data pooled from 6 articles. CI = confidence interval, SD = standard deviation.

In six of the reviewed studies, FES was applied early (less than 6 months) after stroke to prevent or treat subluxation. In all experiments, the two major muscles that counteract shoulder subluxation (supraspinatus and posterior deltoid) were stimulated.  Figure 2 shows the results of the meta-analysis. From the overall 214 participants recruited in all six studies, only one subject did not complete the final assessment, which is negligible compared to the total number of participants. Therefore posttreatment data from 213 subjects were pooled for the meta-analysis. Due to the similarity in the measurement methods, a fixed effect model was used. The analysis of the pooled data indicated a significant improvement in shoulder subluxation in the experimental (FES) group compared to the control group (effect size (*g*) = 0.7, *P* value ≤ 0.00001, and power = 0.99). The results suggest that FES applied in addition to conventional therapy can prevent/reduce shoulder subluxation by 4.9 mm (95% CI: 3.3 to 6.6 mm) compared to conventional therapy alone.

The quality assessment of the articles showed that there is a level 1a evidence from one good quality RCT (Kim-E), and level 2a evidence from five fair quality RCTs (Koyoncu, Baker, Faghri, Linn, Wang-E) on the effect of FES and conventional therapy on shoulder subluxation early after stroke, compared to conventional therapy alone. In all these studies, the electrical stimulation was applied to both supraspinatus and posterior deltoid muscles. In all studies, treatment was done for at least five days a week. Treatment time in all studies was at least four weeks (minimum four and maximum eight weeks). FES was applied through surface electrodes with a frequency ranging from 10 to 36 Hz across the different studies.

#### 3.1.2. Late Treatment

**Figure 3:**
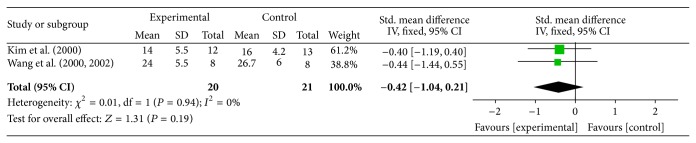
Effect of FES on shoulder subluxation “late” after stroke, with data pooled from 2 articles. CI = confidence interval, SD = standard deviation.

In four studies (Kobayashi-S, Kobayashi-D, Wang-L, and Kim-L) FES was applied late (more than 6 months) after stroke. Except in the experiment by Kobayashi et al. [26], where the deltoid and the supraspinatus were stimulated separately, FES was applied on both muscles in the other two studies. We did not include the quasi-RCT (Kobayashi-S, Kobayashi-D) in the meta-analysis and therefore data from only two studies were pooled. Due to the similarity in the measurement methods a fixed effect model was used.  Figure 3 shows the results of the meta-analysis for the late application of FES. The analysis of the pooled data showed no significant difference between the experimental and control groups (effect size (*g*) = 0.42, *P* value = 0.19, and power = 0.38). FES added to conventional therapy late after stroke could prevent/reduce shoulder subluxation by 2.0 mm compared to the conventional therapy alone.

The quality assessment of articles showed that there is a level of 3 evidence from two poor quality quasi-RCTs (Kobayashi-S and Kobayashi-D) on the effects of FES late after stroke. However, there is a 1a evidence from one good quality RCT (Kim-L) and 2a evidence from one fair quality RCT (Wang-L) showing no significant difference between the experimental and control groups.

#### 3.1.3. Follow-Up

Four studies (Linn, Wang, Faghri, and Baker) performed a follow-up assessment after the termination of FES treatment. In one fair quality study (Linn, level 2a), subjects were reassessed at 4 and 12 weeks after the cessation of treatment. Although the study group had shown a significant reduction in subluxation by the end of the treatment, such an effect was not maintained after the 4th and 12th weeks of follow-up period. No difference was found between the groups at the end of the 12th week. Wang-E (level 2a) reported similar results when reassessing subjects 6 weeks after the termination of the treatment. The significant effect that was seen after the 6 weeks of treatment was not maintained after the 6-week follow-up period. However, when treatment was resumed for another 6 weeks, the same effects of FES were observed again. Baker (level 2a) also reported a reduction in the observed effects after three-month follow-up. However, the authors did not report whether or not the effect of treatment was still significant after the follow-up period. Finally, Faghri (level 2a) reported that the reduced subluxation in the experimental group was still maintained after a 6-week follow-up.

### 3.2. Pain

The level of pain was assessed as an outcome measure in nine studies (Faghri et al. [33], Linn et al. [29], Wang-E, Wang-L [31], Kobayashi-S, Kobayashi-D [26], Mangold et al. [36], Church et al. [34], and Koyuncu et al. [27]). Six studies were done early and three studies late after stroke. In all of these studies, FES was applied either on supraspinatus, posterior deltoid, or both, except in one study (Mangold) where FES was applied on anterior deltoid and triceps brachii. Subjects did not perform any activity nor did they receive any extra treatment during the FES sessions, except in one study (Mangold) where subjects performed reach and grasp movements while being stimulated by FES.

In order to perform a meta-analysis, the studies were categorized based on the methodologies used in the assessment of pain. This yielded two main groups: studies that assessed pain based on the pain-free range of motion (ROM) and studies that assessed subjective pain with numerical scales. A meta-analysis was performed for each group of studies separately. One experiment (Linn et al.) reported data both for pain-free ROM and for numeric scale and was therefore considered in both meta-analyses. From a total of 213 participants who were assessed for pain, 10 participants passed away and did not complete the experiment. Therefore data from 203 participants were pooled for meta-analysis.

#### 3.2.1. Early Treatment

**Figure 4:**
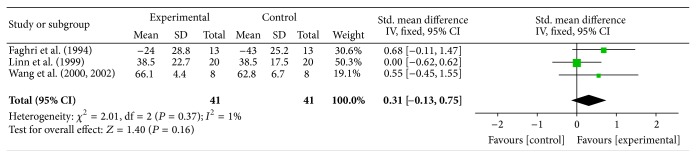
Effect of FES on shoulder pain “early” after stroke, measured as pain-free range of lateral rotation, with data pooled from 3 articles. CI = confidence interval, SD = standard deviation.

Three studies (Faghri, Linn, and Wang-E) assessed pain based on pain-free range of lateral rotation. Posttreatment mean and SD values were pooled for the meta-analysis. Due to the similarity in measurement methods a fixed effect model was used. The result of the meta-analysis showed no significant increase in the pain-free range of lateral rotation in the experimental group compared to the control (effect size (*g*) = 0.31, *P* value = 0.16, and power = 0.47) (Figure 4). On average, the experimental group showed 3.7° increase in the pain-free range of lateral rotation compared to the control group.

**Figure 5:**
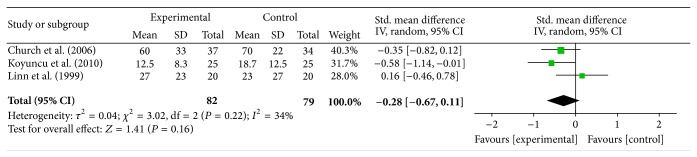
Effect of FES on shoulder pain “early” after stroke, measured with pain numeric scales, with data pooled from 3 articles. CI = confidence interval, SD = standard deviation.

On the other hand, four studies (Church, Koyuncu, Linn, and Mangold) measured the level of pain subjectively using numeric pain scales. One study (Mangold) did not report posttreatment data; therefore only three studies were considered for the meta-analysis. In order to compare the results, all posttreatment data were converted to percentages. Since the scales were similar in concept but differed in the number of points (e.g., 0–6 or 0–10), a random effect model was used. The analysis of the pooled data showed no significant difference between the experimental and control groups (effect size = 0.28, *P* value = 0.16, and power = 0.64) Figure 5.

The quality assessment of the articles showed that, among the studies that measured pain based on pain-free range of lateral rotation, one study (Faghri, level 2a) reported a significant increase in the range of motion without pain, thereby a decrease in the overall level of pain, whereas two studies (Linn, Wang-E, level 2a) showed no significant difference in the pain-free range of lateral rotation between the experimental and control groups.

In the group of studies that measured the level of pain subjectively, there was an absence of evidence of FES effectiveness on shoulder pain, compared to conventional therapy alone, from one good quality RCT (Church, level 1a) and from one fair quality RCT (Koyuncu, level 2a) that used a pain numeric rating scale; from one good quality RCT (Mangold, level 1a) that used the pain scale of the Chedoke-McMaster Assessment; and from one fair quality RCT (Baker, level 2a) that assessed the pain level based on subjects' self-report.

#### 3.2.2. Late Treatment

In two quasi-RCTs with poor quality (Kobayashi-S and Kobayashi-D, level 3) the level of pain was assessed late after stroke using a visual analog scale during active rotation of the shoulder. From the total participants of these two studies (*n* = 17), only seven patients (47%) reported shoulder pain, six in the FES and one in the control group. From the six participants in the FES group, four patients reported a 50% reduction in pain at the end of the treatment, whereas no change was reported for the patient in the control group. The authors did not report if these changes were statistically significant. In addition, Wang-L (level 2a) that measured pain based on the pain-free range of lateral rotation also reported no significant effect of FES on pain when added to conventional therapy, compared to the conventional therapy alone.

### 3.3. Motor Function

Nine studies (Wang-E, Wang-L [31], Church et al. [34], Faghri et al. [33], Linn et al. [29], Nakipoglu et al. [35], Kobayashi-S, Kobayashi-D [26], and Mangold et al. [36]) investigated the effects of FES on motor function after stroke. The retrieved articles were divided into two groups (early or late) based on the time after stroke. In all of the studies FES was applied either on the supraspinatus, the posterior deltoid, or both, except in one study (Mangold) where FES was applied on the anterior deltoid and on the triceps brachii. Similar to the pain outcome, subjects did not perform any activity nor did they receive any extra treatment during the FES sessions, except in one study (Mangold) where subjects performed reach and grasp movements while being stimulated by FES.

#### 3.3.1. Early Treatment

**Figure 6:**
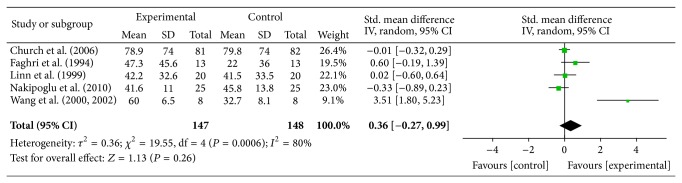
Effect of FES on motor function “early” after stroke, measured with assessment scales, with data pooled from 5 articles. CI = confidence interval, SD = standard deviation.

In six of the nine studies (Mangold, Church, Nakipoglu, Linn, Faghri, and Wang-E), FES was applied early after stroke. All of these studies had used at least one form of assessment scale for the evaluation of upper arm function. In cases where two or more motor function assessment scales were used, the primary scale was considered for the meta-analysis. Since the measures for motor function differed across studies, all data were first converted to percentages for the meta-analysis, where we employed a random effect model. From the six studies in the early treatment group, one study (Mangold) did not report the posttreatment data and was therefore not considered. From a total of 305 participants recruited for the assessment of upper arm motor function, 10 subjects passed away and did not complete the experiment; therefore data from five studies including 295 participants were pooled for meta-analysis. The result showed that adding FES to conventional therapy is not superior to conventional therapy alone in the improvement of arm motor function (effect size = 0.36, *P* value = 0.26, and power = 0.98) Figure 6.

The quality assessment of the articles showed that there is 1a evidence from three good quality RCTs (Church, Mangold, and Nakipoglu) and 2a evidence from one fair quality RCT (Linn) favoring no significant effect of FES therapy in improving motor function of the arm early after stroke, compared to conventional therapy alone. On the contrary, there is 2a evidence from two fair quality RCTs (Faghri, Wang-E) favoring the significant effect of FES therapy.

In the group of articles that showed no superiority of FES over conventional therapy alone for motor function, Linn (level 2a) used the upper arm section of the Motor Assessment Scale [37]; Church (level 1a) used the Action Research Arm Test [38], the Frenchy arm test [39] (these tests are situated at the activity level rather than motor function), and the Motricity index [40]; Mangold (level 1a) used the Chedoke-McMaster Stroke Assessment [41]; and Nakipoglu (level 1a) used the Brunnstrom stage [42] and the Ashworth scale [43] for the assessment of upper arm motor function. In the group of studies that found a significant effect of FES therapy, Wang-E (level 2a) used the Fugl-Meyer Assessment [44] and Faghri (level 2a) used Bobath Assessment Chart [45] and EMG assessment of the deltoid muscle.

#### 3.3.2. Late Treatment

In three (Kobayashi-S, Kobayashi-D, and Wang-L) of the nine retrieved articles, FES was applied late after stroke for the restoration of motor function. Since the measures of motor function were not identical in these studies, no meta-analysis was attempted. The quality assessment of the articles showed that there is a level of evidence of 3 from two poor quality quasi-RCTs (Kobayashi-S, Kobayashi-D) on the effect of FES on motor function compared to conventional therapy alone. The measurement methods in these two studies were abduction force assessment and EMG assessment of the stimulated muscles. On the contrary, there is 2a evidence from one fair quality RCT (Wang-L) showing no superiority of adding FES to the treatment program compared to the conventional therapy alone late after stroke. In this study, motor function was evaluated using the Fugl-Meyer Assessment scale.

## 4. Discussion

### 4.1. Shoulder Subluxation

The results of this review show that the application of FES in addition to conventional therapy is superior to conventional therapy alone in the prevention or treatment of shoulder subluxation early (less than 6 months) but not late (more than 6 months) after stroke. However, the results of the late application of FES should be interpreted with caution because of the low number of studies included as well as the low observed power of the meta-analysis (0.38). These results are consistent with those presented in the review by Ada and Foongchomcheay [15].

One of the considerations regarding the observed effects in the FES group is whether such effects could be due, in part, to the additional therapy sessions that these subjects received, compared to the control group. Indeed, none of the retrieved studies for the subluxation outcome provided an equivalent amount of treatment time to their two groups; FES was always in addition to conventional treatment. On the other hand, subjects in the FES group remained passive; they did not perform any extra activity while receiving FES treatment. Therefore the only additional treatment that the experimental group received was electrical stimulation. We should also note that although subjects in the FES group had 3–5 additional electrotherapy sessions per week compared to the control group, the nature of this type of treatment is not comparable with that of the conventional treatment in the control group. The conventional treatment of shoulder subluxation, especially during the flaccid phase when there is no active contraction of deltoid and supraspinatus, may include using a traditional sling and arm support that prevents shoulder subluxation [32] or employing preventive measures such as early range of motion exercises, proper positioning, and passive support of soft tissue structures. In contrast, FES directly stimulates the nerves of the paralyzed muscles and produces contraction in those muscles. Therefore it seems very unlikely that the additional treatment time could be responsible for the reduction of subluxation in the FES group, compared to the control group. These changes need to be seen as a direct effect of FES.

There have been debates among investigators on the short- and long-term effects of FES in stroke patients. Although most authors agree on the short-term effects of FES [46], not everyone agrees on its long-term effects. Such controversy in the results has been reported in a meta-analysis by Robbins et al. [47]. The results of our review are strongly suggestive of the short-term effects of FES but are inconclusive about the long-term effects. Three studies that performed a follow-up assessment showed no positive long-term effects after the cession of treatment and only one study showed that the effects remained. More good quality studies are needed, in order to reach conclusive results about the long-term effect of FES therapy on shoulder subluxation.

### 4.2. Pain

Price and Pandyan [48] performed a review on the effects of electrical simulation (any type) on shoulder pain after stroke but did not reach any conclusive results due to the low number of good quality RCTs. On the other hand, Ada and Foongchomcheay [15] reported no effect of FES therapy on shoulder pain early (less than 2 months) after stroke but reported a significant effect of FES late (more than 2 months) after stroke. It should be noted however that, in their meta-analysis, the authors pooled data from only 3 studies for the early group and from only 2 studies for the late group.

The results of this review show that FES is not superior to conventional therapy alone for the reduction of shoulder pain. The findings of the two meta-analyses for the studies that measured pain functionally (pain-free range of lateral rotation) and those that measured pain with self-report measurement scales (numerical pain scale, VAS) showed no significant difference between the experimental and control groups. Increase in the pain-free ROM in the shoulder is an indirect indicator of shoulder pain, reported in three RCTs that were carried out in 2002 and earlier. Only one of the three studies showed an improvement in the pain-free ROM. On the other hand, self-report pain assessments are methods that have been used in more recent studies, which can better indicate the patients' overall feeling of pain. However, we should be still cautious about these results because this type of assessment might not be the ideal measurement in stroke patients. For example, Pomeroy and colleagues [49] reported an acceptable level of interrater reliability of the VAS (ICC = 0.62–0.79) in the measurement of shoulder pain after stroke but at the same time reported a consistently large systematic bias between pairs of raters and suggested that training in pain behavior interpretation should be provided to the raters. On the other hand, Price et al. [50] found that many patients after a stroke were unable to successfully complete self-report measurement scales, including the VAS. Therefore any result measured with a self-report scale in stroke patients should be treated with caution. The results of this review are inconclusive about the effect of FES on shoulder pain late after stroke. This is due to a very low number of studies that have applied FES late (more than 6 months) after stroke.

### 4.3. Motor Function

The findings of this review show that FES therapy does not have a significant effect on upper arm motor function early after stroke compared to conventional therapy alone. In the retrieved articles that evaluated motor function early after stroke, the measures of motor function varied greatly. Levin et al. [51] suggest that there should be a distinction between clinical* impairment* and* function* measures.* Impairment* scales measure specific motor aspects that are not related to task accomplishments (spasticity, strength, and isolated joint motion), whereas* functional scales* measure the level of task success (jar opening, key turning) [51]. In this review we found that the group of studies that had reported a significant effect of FES on motor function used methods that measure* impairment*, not* function or activity*. For example Wang-E used the Fugl-Mayer Assessment, which only measures isolated joint motion and is considered as an impairment measure [51]. Similarly, Faghri used EMG activity of stimulated muscles, another measure of impairment, as an indicator of motor function. In fact, these three studies showed that FES increases the activity of the muscles in the arm and therefore increases isolated shoulder joint movements. However, it is not clear if such improvements in muscle activity and joint motion can be translated to improvement in motor function. On the other hand, in the group of studies that have found no superiority of FES over conventional therapy, there are good quality articles that have mostly used those assessment methods that measure* function* or* activity* instead of* impairment*. The Motor Assessment Scale used by Linn, the Action Research Arm Test, Frenchy Arm Test, and Motricity Index used by Church, as well as the Chedoke-McMaster Stroke Assessment used by Mangold, are all examples of functional or activity measures. Therefore, the higher number of articles that favor no effect of FES on upper arm motor function, their higher quality, and the fact that they have assessed motor function with an actual functional measure are all suggestive of no superiority of FES over conventional therapy in the restoration of arm motor function early after stroke.

Only three articles studied the effect of FES therapy late after stroke, from which two studies found a significant effect and one study found no effect. Similar to the early intervention studies, the two articles that reported a significant effect had used EMG activity and arm abduction force and the study that had found no significant effect had used Fugl-Meyer Assessment as their motor function assessment method, which are again not functional measures. Therefore more studies looking at functional measures are needed in order to reach a broad conclusion about the effects of FES on upper arm motor function late after stroke.

## 5. Conclusion

The purpose of this paper was to review the literature that estimated the extent to which FES impacts on shoulder subluxation, pain, and upper arm motor function in stroke patients. The findings suggest that initiating the FES treatment early after the incidence of stroke can significantly reduce the level of shoulder subluxation. Such an effect is mostly observed during the treatment period, not after a follow-up period. More research is still required to examine possible long-term effects of FES therapy on shoulder subluxation. Furthermore, the results of this review suggest that FES does not have any effect on pain compared to conventional therapy alone if applied early after stroke. The results of this review further show that FES therapy early after stroke is not superior to conventional therapy alone in the restoration of motor function in the upper arm. Due to the low number of studies, the results are inconclusive about the FES effectiveness late after stroke.

## Figures and Tables

**Table 1:** Search strategy for Medline database.

Keywords	Medline
(1) Electrical stimulation	122378
(2) Functional electrical stimulation	1483
(3) Neuromuscular electrical stimulation	522
(4) FES	3333
(5) NMES	536
(6) Or/1–5	124932
(7) Stroke	174199
(8) Hemiplegia	13150
(9) Or/7-8	184835
(10) Shoulder joint	14935
(11) Subluxation	7739
(12) Pain	467097
(13) Motor function	13027
(14) Or/10–13	496229
(15) 6 + 9 + 14	229

FES: functional electrical stimulation.

NMES: neuromuscular electrical stimulation.

**Table 2:** Summary of the 10 retrieved articles.

	PEDro, Evidence level	Subjects	Intervention	Outcome measures	Results
Kobayashi et al., 1999 [26]	**3, 3**	*N* = 17 **Mean age:** S-group: 59.3 ± 13.1 D-group: 69.3 ± 7.4 Control: 53.2 ± 9.2	**Conventional treatment:** including neuromuscular facilitation, joint mobilization, muscle stretching of the shoulder + **electrical stimulation** 15 min/session, 2 session/day, 5 days/week, 6 weeks	**Subluxation:** in mm using x-rays **Pain:** VAS during active shoulder abduction **Motor function:** maximal abduction contraction/ EMG measurement of supraspinatus and deltoid	**Subluxation:** decreased in S and D groups compared to control **Pain: **was reduced by 50% in a number of subjects **Motor function: ** increase of abduction force and EMG activity of supraspinatus and deltoid

Koyuncu et al., 2010 [27]	**5, 2a**	*N* = 50 **Mean age:** Study group: 60.7 ± 9.49 Control: 62.0 ± 9.72	**Conventional PT ** **+** **electrical stimulation** 20 min/session, 5 session/day, 4 weeks	**Subluxation:** in mm using x-ray **Pain:** VAS in active and passive shoulder flexion and abduction	**Subluxation: **decreased significantly after treatment **Pain:** no change after treatment

Kim et al., 2000 [28]	**6, 1a**	*N* = 44 **Mean age:** Study group: 55.3 ± 7.3 Control: 58.2 ± 8.1	**Conventional PT and arm sling** + **electrical stimulation** 30 min/day, 5 days/week, 6 weeks	**Subluxation: **in mm using x-ray	**Subluxation:** was prevented and reduced after 6 weeks of FES training in early intervention group. No effectiveness for patients in late intervention group

Linn et al., 1999 [29]	**5, 2a**	*N* = 40 **Mean age:** Study group: 71 Control: 73	**Conventional PT and OT ** + **electrical stimulation,** 30–60 min/session, 4 sessions/day, 4 weeks	**Subluxation:** in cm using X-ray **Pain:** measurement of pain-free range of lateral rotation and Verbal Rating Scale (0–4) **Motor function:** Motor Assessment Scale (0–6) **Arm girth:** in cm to measure muscle bulk	**Subluxation:** decreased after the treatment but was not maintained at the end of follow-up period **Pain:** decreased during the treatment, not maintained after follow-up **Motor function:** no difference between groups **Arm girth:** no difference between groups

Wang et al., 2000, 2002 [30, 31]	**4, 2a**	*N* = 32 **Mean age:** Study group: 56.1 ± 7.4 Control: 56.4 ± 8.4	**Conventional treatment ** **+** **electrical stimulation** 0.5–6 hour/session, 1–3 sessions/day, 5 days/week, 6 weeks	**Subluxation:** in mm using X-ray **Motor function:** Fugl-Meyer Assessment	**Subluxation:** decreased after FES training in hemiplegic subjects with short post-onset duration, but not changed in subjects with subluxation >1 year **Motor function:** increased significantly in patients with short post-onset duration but not in patients with long duration after stroke

Baker and Parker 1986 [32]	**4, 2a**	*N* = 63 **Mean age:** Study group: 56 Control: 55	**Conventional therapy:** conventional hemi sling and wheelchair with arm support + **electrical stimulation** 0.5–7 hour/session, 1–3 sessions/day, 5 days/week, 6 weeks	**Subluxation:** in mm using X-ray **Pain:** subjective self-report	**Subluxation:** decreased in study group after FES treatment. After 3-month follow-up period, the effect of FES was not maintained **Pain:** no reduction in the level of pain was observed

Faghri et al., 1994 [33]	**4, 2a**	*N* = 26 **Mean age:** Study group: 65 ± 13 Control: 69 ± 12	**Conventional therapy ** + **electrical stimulation** 1.5–6 hour/session, 1 session/day, 7 days/week, 6 weeks	**Subluxation: **in cm using x-ray **Pain:** pain-free range of passive external rotation **Motor function:** Bobath assessment chart/EMG assessment of deltoid muscle	**Subluxation:** reduced compared to the control group **Pain:** pain-free passive range of external rotation in shoulder was increased **Motor function:** significantly improved based on Bobath assessment chart. EMG activity of deltoid was significantly increased

Church et al., 2006 [34]	**8, 1a**	*N* = 176 **Mean age:** Study group: 75.5 [64–81] Control 73.5 [65.8–79]	**Conventional therapy ** + **electrical stimulation** 1 hour/session, 3 session/day, 7 days/week, 4 weeks	**Pain:** numerical rating scale **Motor function:** Action Research Arm Test, Frenchy Arm Test, Motricity Index	**Pain:** no significant difference between the groups **Motor function:** no significant difference after 4 weeks of treatment and 3 months after stroke

Nakipoglu et al., 2010 [35]	**6, 1a**	*N* = 60 **Mean age:** Study group: 59.3 ± 14.86 Control: 62.83 ± 12.25	**Conventional therapy ** + **electrical stimulation** 1 hour/session, 1 session/day, 5 days/week, 4 weeks	**Motor function:** Brunsrtrom stages, Ashworth scale	**Motor function:** no significant difference for any of the outcome measures

Mangold et al., 2009 [36]	**6, 1a**	*N* = 23 **Mean age:** Study group: 62 ± 16.2 Control: 57.5 ± 16.7	**Conventional therapy ** + **electrical stimulation** 30 minute/session, 1 session/day, 3 days/week, 4 weeks	**Pain:** pain item of Chedoke McMaster Stroke Assessment **Motor function:** Chedoke-McMaster Stroke Assessment	**Pain:** no significant difference between the groups **Motor function:** no significant difference between the groups

FES: functional electrical stimulation; RCT: randomized control trial; PEDro: physiotherapy evidence database; EMG: electromyography; PT: physiotherapy; OT: occupational therapy; VAS: Visual Analogue Scale.

## Limitations of the Review

The main limitation of this review is that the definitions and assessment methods of outcome measures were not similar across studies. This was especially the case for pain and motor function outcomes. In addition, some of the studies reported median and min/max or median and interquartile instead of mean and standard deviations. Therefore we had to estimate the mean and standard deviations for meta-analyses using the available formulae, assuming a normal distribution of data.

The findings of this review are limited to hemiplegia caused by stroke. Further research is needed to clarify the effect of FES in other types of hemiplegia caused by other neurological conditions.

## Clinical Implications

FES can be added to the conventional therapy for the prevention or reduction of shoulder subluxation by an average of 4.9 mm, early (less than 6 months) after stroke. However, based on the current literature, FES should not be used to reduce pain or improve motor function in the upper arm following stroke.
